# Mindfulness and clinical hypnotherapy in sexual desire disorders: mechanisms, psychophysiological pathways, and integrative therapeutic sequencing

**DOI:** 10.3389/fpsyt.2026.1798650

**Published:** 2026-05-08

**Authors:** Thomas Stompe, Noam Ritter, Wolfgang Kostenwein

**Affiliations:** 1 Department of Psychiatry and Psychotherapy, Medical University of Vienna, Vienna, Austria; 2 Österreichische Gesellschaft für Forensische Psychiatrie, Vienna, Austria; 3 Institut für Sexualpädagogik, Vienna, Austria

**Keywords:** biopsychosocial model, clinical hypnotherapy, expectancy modulation, hypoactive sexual desire disorder, inhibitory sexual regulation, interoception, mindfulness-based intervention, sexual desire disorder

## Abstract

**Background:**

Sexual desire disorders are increasingly conceptualized within biopsychosocial and regulatory models that emphasize inhibitory processes—such as evaluative self-monitoring, maladaptive expectancy, avoidance learning, relational insecurity, and stress dysregulation—rather than primary absence of excitation. Mindfulness-based interventions and clinical hypnotherapy both target core mechanisms implicated in desire inhibition through distinct yet partially overlapping pathways.

**Aims:**

This narrative review synthesizes theoretical models and empirical findings regarding mindfulness-based interventions and clinical hypnotherapy in sexual desire disorders. Particular emphasis is placed on distinguishing between direct randomized evidence and indirect mechanistic support from adjacent medical domains. A mechanism-based sequencing framework for clinical integration is proposed.

**Methods:**

A theory-informed narrative review was conducted using structured literature searches and reference tracking. Extracted study characteristics—including sample composition, study design, intervention format, comparator conditions, and reported outcomes—were organized into summary tables to enhance transparency. Direct evidence (randomized or controlled trials targeting sexual desire disorders) was differentiated from indirect evidence (adjacent hypnosis research addressing expectancy, stress regulation, and attentional modulation).

**Results:**

Randomized controlled trials demonstrate clinically meaningful improvements in sexual desire and distress following mindfulness-based interventions, with small-to-moderate pooled effects reported across studies. In contrast, hypnotherapy-specific randomized trials targeting primary sexual desire disorders remain limited. Available support derives largely from mechanistic alignment with inhibitory models and from adjacent medical domains demonstrating effects on expectancy modulation, distress reduction, and stress-related physiological markers. Psychophysiological findings suggest potential modulation of cortisol and oxytocin dynamics during hypnotic interaction, although causal mediation of sexual outcomes remains unestablished.

**Conclusions:**

Mindfulness-based interventions currently occupy a stronger direct empirical position in the treatment of sexual desire disorders. Clinical hypnotherapy represents a theoretically coherent but underexamined modality whose application rests on mechanistic plausibility and indirect evidence. By explicitly distinguishing evidentiary tiers and summarizing study characteristics in structured tables, this review provides a transparent framework for future hypothesis-driven randomized trials and mediator-focused research evaluating integrative sequencing strategies.

## 1 Introduction

Sexual desire is a multidimensional construct shaped by neurobiological vulnerability, psychological meaning-making, relational dynamics, and sociocultural context (1, 2). Contemporary diagnostic frameworks emphasize that clinically relevant sexual desire disorders must be characterized not only by reduced interest but also by persistent distress and contextual impairment (3, 4). The DSM-5-TR and ICD-11 both caution against pathologizing normative variability in sexual interest, underscoring the importance of relational and situational assessment (3, 4).

Theoretical developments over the past two decades have shifted the understanding of desire from a linear drive model toward interactional and regulatory perspectives. Basson’s model of female sexual response proposed that desire often emerges responsively within contexts of emotional intimacy and safety rather than as a spontaneous biological impulse (5). The Dual Control Model similarly conceptualizes sexual functioning as the dynamic balance between excitatory and inhibitory systems, with heightened inhibition—through anxiety, threat monitoring, shame, or relational insecurity—frequently accounting for diminished desire (6, 7). These models converge in framing low desire as a state of amplified inhibition rather than simple absence of excitation.

Clinical consensus statements further emphasize the heterogeneity of hypoactive sexual desire disorder (HSDD) and related presentations, recommending biopsychosocial assessment and multimodal treatment approaches (1, 8). Psychological contributors may include maladaptive cognitive schemas, performance-related self-monitoring, avoidance learning, trauma-related dissociation, and relational conflict (1, 9). In many patients, desire difficulties are embedded within broader patterns of stress dysregulation, attachment insecurity, or negative expectancy regarding sexual responsiveness (5, 6).

Traditional sex therapy approaches—including sensate focus, cognitive restructuring, and couple-based interventions—have demonstrated benefit across sexual dysfunctions (9, 10). Kaplan’s early triphasic model already highlighted the role of anxiety and inhibitory processes in sexual dysfunction, suggesting that psychotherapeutic techniques aimed at reducing fear and restoring affective engagement may be central to treatment (10). Contemporary behavioral and cognitive-behavioral therapies continue to emphasize attentional retraining, cognitive restructuring, and gradual exposure to avoided stimuli (9, 11). However, clinical experience and empirical findings indicate that insight and cognitive reframing alone may not always translate into embodied shifts in sexual responsiveness, particularly when inhibitory processes are affectively entrenched or relationally conditioned (1, 5).

Within this context, experiential and attentional interventions have gained increasing interest. Mindfulness-based approaches seek to reduce evaluative self-monitoring and cognitive distraction while enhancing present-moment awareness and interoceptive tolerance (12, 13). Randomized controlled trials have demonstrated that mindfulness-based cognitive therapy adapted for female sexual interest/arousal disorder can improve desire and reduce sexual distress (12, 14). Meta-analytic evidence suggests small-to-moderate effects across sexual functioning domains, though methodological heterogeneity remains substantial (13).

Clinical hypnotherapy represents a second modality targeting attentional organization and experiential processing. Beyond its well-established efficacy in procedural distress and pain management (15), contemporary hypnosis integrates cognitive, psychodynamic, and relational models (16–18). Experimental research suggests that hypnotic processes can influence expectancy, salience attribution, and potentially stress-related physiological pathways (19, 20). Despite this broader literature, systematic evaluation of hypnotherapy specifically for sexual desire disorders remains limited, and existing applications are often embedded within integrative therapeutic frameworks.

Given the overlapping yet distinct mechanisms of mindfulness and hypnotherapy—meta-awareness and acceptance on the one hand, focused absorption and experiential restructuring on the other—a mechanism-based integrative perspective may offer clinically useful guidance. Rather than positioning these modalities as competing schools, they may be conceptualized as complementary attentional technologies addressing different facets of desire inhibition. The present review therefore synthesizes theoretical and empirical evidence across both approaches, critically evaluates their current evidentiary status, and proposes a pragmatic sequencing model to inform future randomized trials and individualized clinical application.

## 2 Methods

### 2.1 Review design

This article was conducted as a theory-informed narrative review with a mechanism-focused analytic framework. Narrative methodology was selected because the existing literature on mindfulness and hypnotherapy in sexual desire disorders is heterogeneous in diagnostic definitions, intervention formats, comparator conditions, and outcome measurement, limiting the feasibility of quantitative meta-analytic aggregation (9, 13).

Rather than estimating pooled effect sizes, the aim was to synthesize conceptual models, randomized and controlled clinical evidence, and mechanistic hypotheses relevant to inhibitory processes in sexual desire disorders. The review follows established guidance for narrative syntheses while incorporating structured evidence summarization to enhance transparency (21).

### 2.2 Literature search strategy

A focused literature search was conducted using PubMed as the primary database. Search terms included combinations of:

- “sexual desire disorder”
- “hypoactive sexual desire disorder”
- “sexual interest/arousal disorder”
- “mindfulness”
- “mindfulness-based cognitive therapy”
- “hypnosis”
- “hypnotherapy”
- “ego-state therapy”
- “expectancy”
- “interoception”
- “stress regulation”
- “oxytocin”
- “cortisol”

Searches were limited to peer-reviewed articles published in English. Priority was given to randomized controlled trials, systematic reviews, meta-analyses, and consensus statements directly addressing sexual desire disorders. In areas where disorder-specific trials were limited (particularly hypnotherapy), adjacent clinical and experimental literatures were included to evaluate mechanistic plausibility.

Backward reference tracking of key reviews and foundational theoretical papers was performed to ensure coverage of influential models (1, 2, 5–7, 13–16, 19, 20, 22–27).

### 2.3 Study selection and data extraction

Studies were selected based on relevance to one of three domains:

1. Direct clinical trials targeting sexual desire disorders
2. Psychological intervention trials reporting sexual desire–relevant outcomes
3. Adjacent hypnosis research addressing expectancy, stress regulation, or psychophysiological modulation

For each included study, the following variables were extracted:

- Sample characteristics (size, gender distribution, clinical diagnosis)
- Study design (randomized controlled trial, controlled trial, pilot, experimental)
- Intervention format and duration
- Comparator condition
- Outcome domains (desire, sexual distress, relational variables)
- Reported effect sizes or statistical significance (where available)
- Follow-up duration

Extracted data were organized into structured summary tables (**Tables 1**, **2**) to enhance transparency regarding study characteristics, methodological strength, and the distinction between direct and indirect evidence bases.

**Table 1:** Mindfulness-based interventions with desire-relevant outcomes (direct evidence base).

Study	Population / Setting	Design	Intervention	Comparator	Outcomes (desire-related)	Key findings	Notes / Limitations
Brotto & Basson (28)	Women with sexual desire difficulties	RCT	4-session group mindfulness-based therapy	Delayed treatment control	Sexual desire (primary), broader sexual function	Significant improvements in desire and sexual function vs control	Short follow-up; female, Western sample
Brotto et al. (14)	Women with FSAD / interest-arousal disorder	RCT	MBCT adapted for sexual interest/arousal	Control condition (trial-specific)	Desire, distress	Clinically meaningful improvements; mechanism discussion (attention/interoception)	Sample and measures heterogeneous across studies
Paterson et al. (12)	Women with SI/AD	Clinical trial / adapted MBCT	MBCT-adapted protocol	(varies)	Desire, distress	Improvements reported	Often smaller samples
Brotto et al. (29)	Women w/ desire/arousal complaints	Pilot (group psychoeducation w/ mindfulness component)	Mindfulness-based group psychoeducational program	(uncontrolled / pre-post)	Desire/arousal complaints	Preliminary support; mindfulness singled out by participants	Not RCT; early-stage evidence
Brotto et al. (30)	Gynecologic cancer survivors w/ sexual dysfunction	Controlled trial	Brief mindfulness-based CBT intervention	Wait-list control	Sexual functioning incl. desire	Significant improvements vs wait-list	Cancer population; generalizability limits
Banbury et al. (13)	Mixed sexual difficulties	Systematic review/meta-analysis	MBIs across studies	Active/passive controls	Desire & function domains	Small–moderate pooled effects; heterogeneity high	Outcome definitions vary

**Table 2:** Hypnosis/hypnotherapy evidence relevant to sexual desire: direct vs. indirect evidence.

Evidence tier	Study	Population/ Setting	Design	Intervention	Comparator	Relevant outcomes	Key findings	Why it matters for desire
Direct (sexual-domain)	Barton et al. (31)	Breast/gynecologic cancer survivors	Phase II RCT	Hypnosis (3 sessions + home practice)	Progressive muscle relaxation	Sexual interest/satisfaction (secondary), body image	Sexual interest/satisfaction improved more in hypnosis arm (secondary)	Shows *sexual outcomes* can move under hypnosis in an RCT context
Indirect (mechanistic/adjacent)	Olendzki et al. (32)	Elevated stress (college sample)	RCT pilot	Mindful hypnotherapy (8 weeks)	Wait-list	Stress, mindfulness	Feasible; improved stress/mindfulness	Trial template for integrated protocol; mediator logic
Indirect (clinical adjacent)	Grégoire et al. (33)	Posttreatment cancer patients	RCT + 1y follow-up	Group self-care + self-hypnosis	Wait-list	Fatigue, sleep, distress	Reduced fatigue/sleep problems/distress; effects maintained	Supports stress-regulation pathway relevant to desire inhibition
Psychophysiology	Varga & Kekecs (19)	Healthy volunteers	Lab study	Hypnotic interaction	Control/within-subject	Oxytocin, cortisol	Changes relate to relational experience	Biological plausibility: attachment/stress axis
Psychophysiology	Kasos et al. (34)	Active-alert hypnosis context	Experimental	Active-alert hypnosis	Within-subject	Oxytocin, cortisol	Oxytocin ↑ / cortisol ↓ patterns reported	Stress modulation plausibility; cautious interpretation
Contextual review	Kraft & Kraft (35)	Sexual disorders (broad)	Narrative review	Hypnotherapy applications	—	Sexual dysfunctions	Summarizes clinical use across disorders	

### 2.4 Evidence hierarchy and extrapolation framework

Given the disparity in disorder-specific evidence between mindfulness-based interventions and hypnotherapy, an explicit evidentiary distinction was maintained throughout the analysis.

- Direct evidence refers to randomized or controlled trials explicitly targeting sexual desire disorders.
- Indirect evidence refers to randomized or experimental studies in adjacent medical domains where mechanisms relevant to sexual inhibition—such as expectancy modulation, stress regulation, or attentional reorganization—were examined.

The inclusion of indirect evidence does not imply equivalence of efficacy across domains. Rather, such evidence was considered hypothesis-generating and mechanistically informative. Extrapolation was therefore limited to shared regulatory processes rather than assumed clinical generalizability.

### 2.5 Methodological limitations

As a narrative synthesis, this review does not provide pooled effect size estimation and remains subject to potential selection bias despite structured search procedures (21). The absence of disorder-specific randomized trials for hypnotherapy represents a central empirical limitation. Additionally, variability in outcome definitions, short follow-up periods, and predominantly female Western samples constrain generalizability (1, 13).

The proposed integrative sequencing model should therefore be regarded as a clinically informed framework requiring empirical validation through adequately powered randomized controlled trials incorporating active comparators and mediator analyses.

## 3 Results

### 3.1 Historical and conceptual background: from attentional training to experiential modulation

Mindfulness-based interventions and clinical hypnotherapy emerge from distinct historical traditions yet converge in their contemporary clinical application as methods for modulating attention, affect regulation, and embodied meaning.

Mindfulness derives from contemplative traditions in South and Southeast Asia, where sustained attention and non-judgmental awareness were embedded within broader ethical and psychological systems. In Western clinical contexts, mindfulness was operationalized as a secular therapeutic skill aimed at reducing suffering through attentional stabilization, decentering, and emotion regulation (22). The development of mindfulness-based stress reduction (MBSR) and mindfulness-based cognitive therapy (MBCT) translated contemplative practices into structured, manualized interventions suitable for psychiatric and medical populations (22, 23). Empirical research has demonstrated that mindfulness training can reduce rumination, enhance interoceptive awareness, and improve emotion regulation across diverse clinical conditions (24).

Within sexual medicine, mindfulness-based adaptations were introduced in response to findings that cognitive distraction, evaluative self-monitoring, and avoidance of bodily sensation frequently interfere with sexual arousal and desire (12, 14). By shifting attention from performance-based evaluation toward present-moment sensory experience, mindfulness interventions aim to reduce inhibitory overcontrol and restore embodied responsiveness (5, 6, 13).

Clinical hypnotherapy, in contrast, developed within European medical and psychological traditions and evolved through phases of suggestion-based practice, hypnoanalysis, and integrative psychotherapeutic models (17, 25). Modern conceptualizations define hypnosis as a contextually shaped state of focused absorption characterized by enhanced responsiveness to suggestion and flexible salience attribution rather than passive compliance (20, 26). Social-cognitive theories emphasize expectancy and attentional modulation (26), whereas psychodynamic and ego-state models conceptualize hypnosis as facilitating access to dissociated experiential material and internal reorganization (17, 18).

Across medical contexts, hypnosis has demonstrated efficacy in reducing procedural distress and pain perception (15, 27). Neurocognitive models indicate altered functional connectivity in attentional and salience-processing networks during hypnotic suggestion (20), supporting its interpretation as modulation of regulatory processes rather than as a unitary altered state.

Although mindfulness and hypnosis differ phenomenologically—open monitoring versus focused absorption—both may be conceptualized as attentional technologies capable of reorganizing inhibitory processes central to sexual desire disorders.

### 3.2 Mechanisms and “qualities of action”

#### 3.2.1 Mindfulness: meta-awareness, interoceptive tolerance, and reduction of inhibitory overcontrol

Mindfulness-based interventions cultivate non-judgmental present-moment awareness and decentering from cognitive evaluation (22, 23). Mechanistic models propose that mindfulness operates through enhanced attentional regulation, increased body awareness, improved emotion regulation, and altered self-referential processing (24). These mechanisms are directly relevant to sexual desire disorders, where excessive evaluative self-monitoring and cognitive distraction frequently inhibit arousal and desire (5, 6).

Randomized trials summarized in **Table 1** demonstrate that mindfulness-based cognitive therapy adapted for sexual interest/arousal disorder yields significant improvements in sexual desire and reductions in distress (12, 14, 28–30). Proposed mediators include reduced cognitive distraction, increased sensory awareness, and improved emotion regulation. Importantly, mindfulness does not “produce” desire directly; rather, it reduces inhibitory overcontrol consistent with the Dual Control Model (6, 7). By restoring attentional flexibility and interoceptive tolerance, mindfulness may create conditions under which desire can re-emerge.

#### 3.2.2 Clinical hypnotherapy: focused absorption, schema modification, and psychophysiological modulation

Hypnotherapy operates through focused absorption and guided experiential modulation (16, 26). Response expectancy theory suggests that hypnotic suggestion modifies anticipatory schemas and salience attribution (26). In sexual desire disorders, entrenched inhibitory expectancies may perpetuate diminished responsiveness (5, 6).

Beyond expectancy, integrative models situate hypnosis within psychodynamic and ego-state frameworks, facilitating affect integration and reorganization of internal representations (17, 18). Cognitive hypnotherapy further integrates hypnotic absorption with cognitive restructuring processes (16), potentially enhancing modification of maladaptive core beliefs relevant to sexual inhibition.

Evidence summarized in **Table 2** distinguishes between direct and indirect domains. Disorder-specific RCT evidence remains limited (31), whereas robust data from adjacent medical contexts demonstrate reductions in distress and modulation of attentional and affective processing (15, 27). Emerging psychophysiological studies report alterations in cortisol and oxytocin dynamics during hypnotic interaction (19, 34). While preliminary and heterogeneous, these findings align with stress-regulatory models of sexual inhibition (5, 6). However, biological plausibility does not equate to confirmed mediation of sexual desire outcomes. Thus, hypnotherapy in sexual desire disorders currently rests on mechanistic coherence and indirect empirical support rather than disorder-specific randomized confirmation.

### 3.3 Clinical evidence in sexual desire disorders

Structured comparison of the available evidence is presented in **Tables 1**, **2**.

Mindfulness-based interventions demonstrate direct randomized support for improvements in sexual desire and distress (12, 14, 28–30). Meta-analytic findings indicate small-to-moderate pooled effects across sexual functioning domains, though heterogeneity in design and outcome definitions persists (13). Hypnotherapy-specific RCTs directly targeting sexual desire disorders are lacking. Limited controlled evidence from oncology populations suggests improvements in sexual interest as secondary outcomes (31), but generalizability remains uncertain. The asymmetry reflected in **Tables 1**, **2** underscores the need for cautious interpretation. Mindfulness currently occupies a stronger empirical position, whereas hypnotherapy represents a theoretically coherent but underexamined intervention within this population.

### 3.4 Evidence from adjacent medical fields

Evidence from adjacent medical domains, summarized in **Table 2**, demonstrates consistent hypnotic effects on procedural distress, pain perception, and expectancy modulation (15, 27). Randomized pilot trials integrating hypnosis and mindfulness show feasibility and stress reduction effects (36), while oncology trials combining self-care and self-hypnosis report sustained improvements in fatigue, sleep, and distress (37). Such findings support mechanistic plausibility regarding stress regulation and attentional modulation but cannot substitute for disorder-specific randomized trials in sexual medicine.

## 4 Discussion

The present review examined mindfulness-based interventions and clinical hypnotherapy within a shared mechanism-based framework for sexual desire disorders. By explicitly distinguishing between direct and indirect evidence bases (**Tables 1**, **2**), the analysis aimed to clarify both the empirical strengths and the current limitations of these approaches.

### 4.1 Desire as inhibition: integrating regulatory models

Contemporary models of sexual desire increasingly conceptualize diminished desire not as a primary deficit of excitation but as the result of amplified inhibitory processes (1, 5, 6). Basson’s responsive desire model emphasizes relational safety and emotional intimacy as preconditions for sexual engagement (5), whereas the Dual Control Model describes sexual functioning as the dynamic balance between excitatory and inhibitory systems (6, 7). Within this framework, anxiety, shame-based monitoring, relational insecurity, trauma-related conditioning, and chronic stress may heighten inhibitory pathways.

Mindfulness-based interventions directly target such inhibitory overcontrol. As reflected in **Table 1**, randomized trials demonstrate that mindfulness training reduces cognitive distraction, evaluative self-monitoring, and sexual distress (12, 14, 28–30). Mechanistically, mindfulness strengthens meta-awareness and interoceptive tolerance (22, 24), potentially weakening avoidance learning and restoring embodied presence. Importantly, mindfulness does not act as a direct libido-enhancing technique; rather, it reduces inhibitory constraints, consistent with regulatory models of sexual functioning (6).

Clinical hypnotherapy, conceptualized within integrative psychotherapeutic frameworks, may complement this mechanism by targeting entrenched expectancy schemas and emotionally encoded inhibitory beliefs (16–18). As summarized in **Table 2**, disorder-specific randomized evidence remains limited, yet mechanistic alignment with expectancy modulation and stress regulation provides conceptual coherence (15, 27). Hypnosis may facilitate corrective emotional learning through focused absorption, imagery-based restructuring, and enhanced cognitive-affective integration.

Emerging psychophysiological findings reporting modulation of cortisol and oxytocin dynamics during hypnotic interaction (19, 34) offer additional biological plausibility. While preliminary and heterogeneous, these findings align with stress-attachment models relevant to sexual inhibition (5, 6). However, no study to date has established a causal pathway linking hypnotically induced neuroendocrine changes to improvements in sexual desire. Accordingly, biological mechanisms should be regarded as hypothesis-generating rather than empirically confirmed mediators.

### 4.2 Evidentiary asymmetry and epistemic caution

A central finding of this review is the evidentiary asymmetry between mindfulness-based interventions and hypnotherapy in sexual desire disorders. **Table 1** demonstrates that mindfulness is supported by randomized controlled trials directly targeting desire-related outcomes (12, 14, 28–30), with meta-analytic evidence indicating small-to-moderate pooled effects (13). Although methodological heterogeneity persists, the empirical foundation is established.

In contrast, **Table 2** illustrates that hypnotherapy-specific randomized trials targeting primary sexual desire disorders are lacking. Limited controlled data from oncology contexts report improvements in sexual interest as secondary outcomes (31), but generalizability remains uncertain. Most of the evidence supporting hypnosis derives from adjacent domains involving procedural distress, expectancy modulation, and stress regulation (15, 27, 36, 37).

This asymmetry necessitates epistemic caution. Indirect evidence supports mechanistic plausibility but cannot substitute for disorder-specific efficacy trials. The present review therefore does not claim equivalence of empirical support between modalities. Instead, it proposes a structured framework for future hypothesis-driven investigation.

### 4.3 Integrative sequencing: a pragmatic clinical proposal

Within a mechanism-based perspective, mindfulness and hypnotherapy may represent complementary intervention strategies rather than competing schools. Mindfulness emphasizes open monitoring and acceptance, reducing evaluative self-surveillance and enhancing interoceptive tolerance (22, 24). Hypnotherapy emphasizes focused absorption and experiential restructuring, potentially modifying entrenched inhibitory schemas and expectancy patterns (16, 26). A pragmatic sequencing model may therefore be considered. Patients presenting primarily with cognitive overcontrol, performance anxiety, or generalized emotional avoidance may benefit initially from mindfulness-based stabilization. Those with rigid negative expectancy, trauma-related dissociation, or affectively encoded inhibitory schemas may subsequently benefit from adjunctive hypnotherapeutic interventions. This sequencing model remains conceptual and requires empirical validation. Future randomized trials could directly compare mindfulness-first versus hypnosis-first stepped-care designs and incorporate mediator analyses assessing attentional regulation, expectancy change, avoidance reduction, and stress physiology.

### 4.4 Diversity, generalizability, and research priorities

Both **Tables 1**, **2** reveal demographic limitations within the existing literature. Samples are predominantly female, frequently drawn from Western populations, and often limited to heterosexual relationship contexts (1, 12–14). Cultural conceptions of desire, relational norms, and gendered sexual scripts may significantly influence both symptom expression and treatment responsiveness.

Future research should prioritize:

- Inclusion of men and gender-diverse individuals
- Culturally diverse samples beyond Western clinical settings
- Explicit assessment of relational context and attachment variables
- Standardized outcome definitions for sexual desire
- Longer-term follow-up assessments

Additionally, adequately powered randomized controlled trials directly evaluating hypnotherapy for primary sexual desire disorders are needed to clarify both efficacy and mechanism specificity.

## 5 Conclusion

Sexual desire disorders arise from complex interactions among attentional regulation, expectancy processes, relational dynamics, and embodied stress physiology (1, 5, 6). Mindfulness-based interventions demonstrate direct randomized support for improving sexual desire and reducing distress through attenuation of evaluative self-monitoring and enhancement of interoceptive awareness (12, 14, 28–30).

Clinical hypnotherapy, situated within integrative psychotherapeutic models, offers a theoretically coherent approach to modifying inhibitory schemas and expectancy-driven processes. Evidence from adjacent medical domains supports mechanistic plausibility regarding stress regulation and attentional modulation (15, 19, 27, 34, 36, 37). However, disorder-specific randomized controlled trials are required before definitive efficacy claims can be made.

By distinguishing explicitly between direct and indirect evidence bases (**Tables 1**, **2**), the present review aims to provide a transparent and balanced framework for clinical consideration and future research design. A mechanism-based integration of mindfulness and hypnotherapy may ultimately enhance individualized treatment planning, provided that empirical validation continues to advance.
